# Qualitative and quantitative MRI analysis of alveolar soft part sarcoma: correlation with histological grade and Ki-67 expression

**DOI:** 10.1186/s13244-024-01687-8

**Published:** 2024-06-13

**Authors:** Junhui Yuan, Deshun Xie, Shaobo Fang, Fan Meng, Yue Wu, Dongqiu Shan, Nannan Shao, Bangmin Wang, Zhichao Tian, Yuanyuan Wang, Chunmiao Xu, Xuejun Chen

**Affiliations:** 1 Department of Medical Imaging, The Affiliated Cancer Hospital of Zhengzhou University & Henan Cancer Hospital, Zhengzhou, Henan China; 2 Department of Radiology, Heze Municipal Hospital, Heze, Shandong China; 3 Department of Medical Imaging, Zhengzhou University People’s Hospital & Henan Provincial People’s Hospital, Academy of Medical Sciences, Zhengzhou University, Zhengzhou, Henan China; 4 Department of Bone and Soft Tissue, The Affiliated Cancer Hospital of Zhengzhou University & Henan Cancer Hospital, Zhengzhou, Henan China; 5 Department of Pathology, The Affiliated Cancer Hospital of Zhengzhou University & Henan Cancer Hospital, Zhengzhou, Henan China

**Keywords:** Alveolar soft part sarcoma, Magnetic resonance imaging, Histological grading, Ki-67 expression

## Abstract

**Objective:**

To investigate the correlation between MRI findings and histological features for preoperative prediction of histological grading and Ki-67 expression level in alveolar soft part sarcoma (ASPS).

**Methods:**

A retrospective analysis was conducted on 63 ASPS patients (Jan 2017–May 2023). All patients underwent 3.0-T MRI examinations, including conventional sequences, dynamic contrast-enhanced scans with time-intensity curve analysis, and diffusion-weighted imaging with apparent diffusion coefficient (ADC) measurements. Patients were divided into low-grade (histological Grade I) and high-grade (histological Grade II/III) groups based on pathology. Immunohistochemistry was used to assess Ki-67 expression levels in ASPS. Statistical analysis included chi-square tests, Wilcoxon rank-sum test, binary logistic regression analysis, Spearman correlation analysis, and receiver operating characteristic curve analysis of various observational data.

**Results:**

There were 29 low-grade and 34 high-grade patients (26 males and 37 females) and a wide age range (5–68 years). Distant metastasis, tumor enhancement characteristics, and ADC values were independent predictors of high-grade ASPS. High-grade ASPS had lower ADC values (*p* = 0.002), with an area under the curve (AUC), sensitivity, and specificity of 0.723, 79.4%, and 58.6%, respectively, for high-grade prediction. There was a negative correlation between ADC values and Ki-67 expression (*r* = −0.526; *p* < 0.001). When the cut-off value of ADC was 0.997 × 10^−3^ mm²/s, the AUC, sensitivity, and specificity for predicting high Ki-67 expression were 0.805, 65.6%, and 83.9%, respectively.

**Conclusion:**

Qualitative and quantitative MRI parameters are valuable for predicting histological grading and Ki-67 expression levels in ASPS.

**Critical relevance statement:**

This study will help provide a more nuanced understanding of ASPS and guide personalized treatment strategies.

**Key Points:**

- There is limited research on assessing ASPS prognosis through MRI.
- Metastasis, enhancement, and ADC correlated with histological grade; ADC related to Ki-67 expression.
- MRI provides clinicians with valuable information on ASPS grading and proliferation activity.

**Graphical Abstract:**

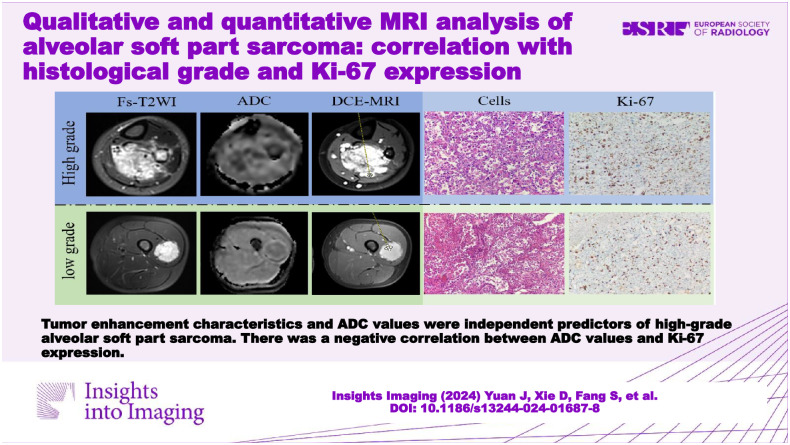

## Introduction

Alveolar soft part sarcoma (ASPS), accounting for about 0.4–1% of soft tissue sarcomas (STSs), is a mesenchymal-origin tumor predominantly affecting young females, typically aged between 15 and 35 years [1–3]. It presents as a painless, slowly growing mass, notable for its rich vascularity, high propensity for early metastasis, poor prognosis, and resistance to conventional cytotoxic chemotherapy [4]. The histological grading of ASPS, which classifies tumors into Grades I, II, and III based on cell differentiation, necrosis extent, and mitotic rate, serves as a key prognostic factor [5]. Studies often categorize Grades II and III together as high-grade, associated with significantly higher mortality compared to Grade I [6]. The proliferation marker Ki-67 is particularly crucial, with high expression levels linked to increased mortality, recurrence rates, and adverse outcomes [7].

Despite its clinical significance, diagnosing and grading ASPS remains challenging. Percutaneous biopsy, the standard preoperative diagnostic approach, carries risks like tumor cell dissemination [8]. Moreover, due to the tumor’s heterogeneity, biopsies may yield inconsistent results, potentially leading to suboptimal treatment decisions. Therefore, there is an urgent need for a non-invasive, accurate method to differentiate ASPS histological grades. Magnetic resonance imaging (MRI) has emerged as a promising tool in this regard, capable of reflecting some clinical and pathological characteristics of ASPS. Typical MRI findings include slightly higher signal intensity on T1-weighted images (T1WI) and high signal intensity on T2-weighted images (T2WI) due to the tumor’s rich blood supply, which also manifests as tortuous and dilated vascular structures [2]. Intratumoral necrosis, infiltrative growth patterns, and tumor vascular cavities were discernible in MRI scans to aid in differentiating ASPS from other STSs [9].

The existing literature on ASPS is limited, primarily comprising case reports with minimal in-depth investigation into the prognostic evaluation of ASPS through imaging. This gap underscores the need for comprehensive studies to explore the correlation between MRI features and ASPS histological grading and Ki-67 expression levels. This study aims to summarize the clinical and MRI manifestations and histopathological features of ASPS, seeking to predict histological grading and Ki-67 expression levels preoperatively. By providing additional imaging-based insights, this research could significantly assist in ASPS diagnosis and clinical decision-making, offering a more nuanced understanding of ASPS.

## Materials and methods

This study received approval from the Medical Ethics Committee of Henan Cancer Hospital (Approval No.: 2023-KY-0106) and was exempt from the requirement for informed consent. A retrospective data collection was conducted on 63 ASPS patients who underwent surgical pathological confirmation and were enrolled between January 2017 and May 2023 at three hospital centers. Inclusion criteria were: (I) all patients were histologically diagnosed with ASPS; (II) patients had not received any form of treatment (including surgery, radiotherapy, chemotherapy, or targeted therapy) prior to admission; (III) patients had complete medical records, including detailed clinical data, previous imaging results, and histopathology reports; (IV) the interval between surgery and MRI examination was less than one month. Exclusion criteria were: (I) patients with a history of other types of tumors or major diseases; (II) patients with contraindications for MRI examination; (III) patients who had received treatment that could potentially affect MRI or diffusion-weighted imaging (DWI) features; (IV) patients lacking sufficient MRI, DWI, or histopathological data.

### MRI examination protocol

MRI scans were performed on 63 patients using a PHILIPS Ingenia 3.0MR scanner, which included both conventional imaging and dynamic contrast-enhanced (DCE) scans. The conventional imaging sequences consisted of T1-weighted imaging with turbo spin echo (T1WI-TSE) (TR: 643.96 ms, TE: 13.97 ms), T2-weighted imaging with turbo spin echo (T2WI-TSE) (TR: 2843.0 ms, TE: 129.84 ms), fat-suppressed T2-weighted imaging with turbo spin echo (fat-suppressed T2WI-TSE) (TR: 1616 ms, TE: 90 ms), and DWI with *b* values of 0 and 800 s/mm². The DCE-MRI scan sequence used fat-suppressed T1-weighted imaging with turbo spin echo (fat-suppressed T1WI-TSE). Gadolinium-based contrast agent was intravenously injected through the elbow vein using a high-pressure injector at a dose of 0.1 mmol per kilogram of body weight, with an injection rate of 2.5 mL/s.

### MR analysis

Qualitative and quantitative MRI features of all cases of ASPS were assessed using a double-blind approach by two radiologists with over 10 years of diagnostic experience. The assessments included site, morphology, size, margin, peritumoral edema, intratumoral necrosis (the imaging features of tumor necrosis on MRI include low signal on T1WI, high signal on T2WI, non-enhancement areas on DCE-MRI, and high signal on DWI), interpretation of T1WI, T2WI signals (the features of high signal on T1WI and low signal on T2WI were defined as an increase and decrease, respectively, relative to the muscle signal on the corresponding sequences), DWI signals, tumor enhancement degree, and time-intensity curve (TIC) type. For the measurement of the apparent diffusion coefficient (ADC), five regions of interest (ROIs) were randomly selected within the tumor on the slice with the maximum tumor dimension (ROI area: 30–80 mm²), avoiding areas of cystic necrosis. The average ADC value was calculated, and the final ADC value was determined as the mean of the measurements from the two radiologists. Intra-class correlation coefficients (ICC) were used to assess the consistency between the two observers for the analysis of conventional MRI features and DWI parameter measurements (ICC < 0.40 indicates poor consistency; 0.41–0.59 indicates fair consistency; 0.60–0.74 indicates good consistency; 0.75–1.00 indicates excellent consistency) [10]. In cases of disagreement between the two radiologists’ imaging analyses, a consensus was reached through discussion.

### Pathological and immunohistochemical evaluation

Histological grading and Ki-67 expression assessment of all tumors were conducted in a double-blind manner by two pathologists with over 10 years of diagnostic experience. The evaluation included assessing the degree of tumor cell differentiation, the number of mitotic figures, and the area of visible tumor necrosis under the microscope. Tumors were classified into Grades I, II, and III based on the Fédération Nationale des Centres de Lutte Contre le Cancer (FNCLCC) system for sarcoma grading, the analysis included cell morphology, nuclear division activity, degree of necrosis and microvessel density, with Grade I categorized as the low-grade group and Grades II and III grouped into the high-grade group. Ki-67 expression was considered low when Ki-67 < 30% and classified as high when Ki-67 ≥ 30% [11].

### Statistical analysis

Data were subjected to statistical analysis using software including SPSS 24.0, Prism 9, and MedCalc. Continuous variables were expressed as mean ± standard deviation (*x* ± *s*) if they followed a normal distribution. If the data did not meet the criteria for a normal distribution, continuous variables were represented by the median (interquartile range). To compare clinical, pathological, and MRI features between the high-grade and low-grade ASPS patient groups, categorical variables were analyzed using the chi-squared test, while continuous variables were analyzed using either the Wilcoxon rank-sum test or independent samples *t* test, depending on the data distribution. Binary logistic regression analysis was employed to identify independent factors predicting high-grade ASPS patients, and the results were presented as odds ratios with corresponding 95% confidence intervals (CIs). Pearson correlation analysis was used for normally distributed and homoscedastic continuous variables, whereas Spearman correlation analysis was employed for non-normally distributed or heteroscedastic variables. Receiver operating characteristic (ROC) curve analysis was performed for observation parameters with statistical significance, with the area under the curve (AUC), sensitivity, and specificity used to evaluate the diagnostic performance for distinguishing high-grade from low-grade ASPS. A significance level of *p* < 0.05 was considered statistically significant.

## Results

### General clinical, pathological, and MRI data analysis

**Fig. 1:**
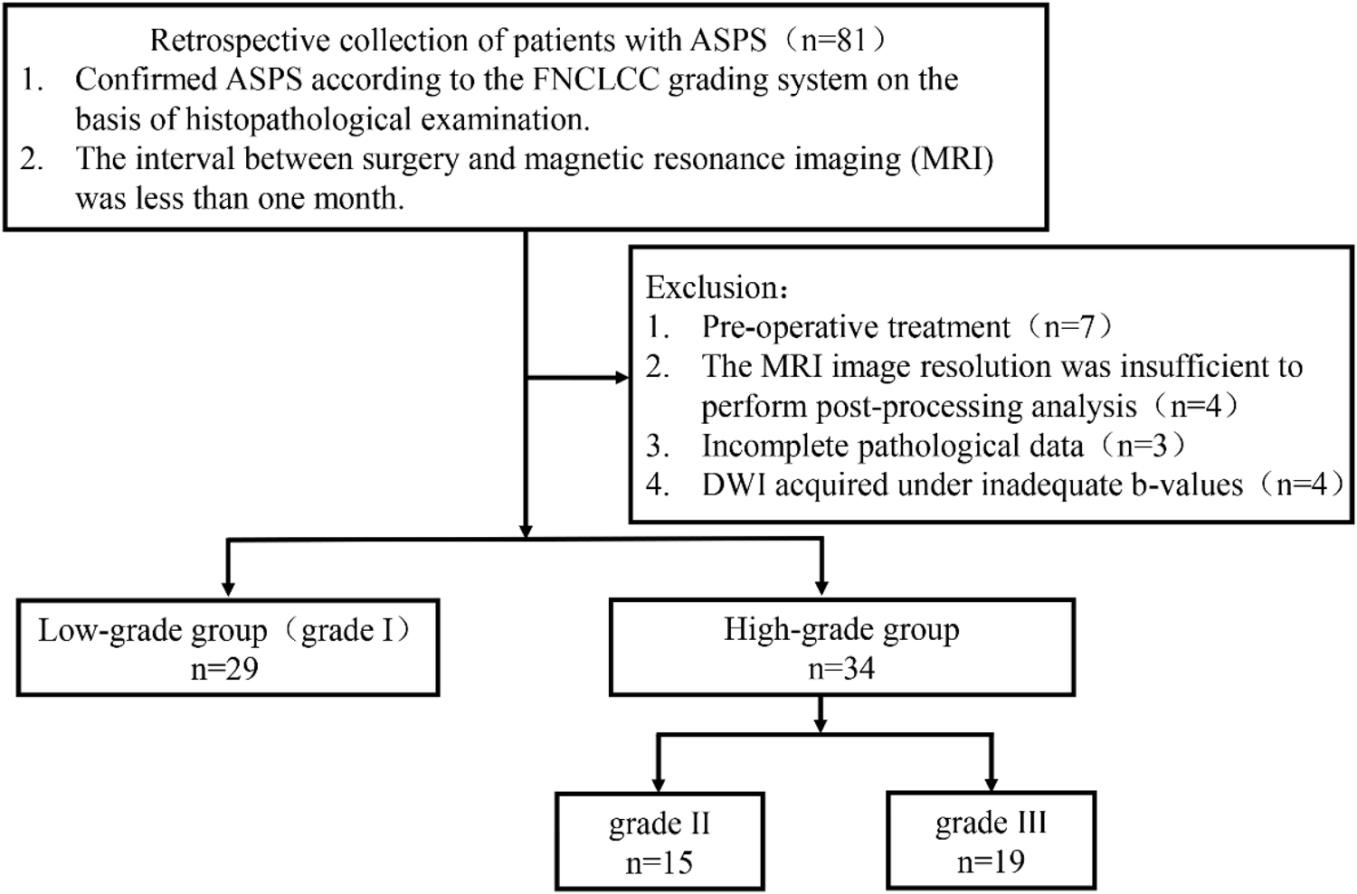
Case selection process flowchart

Among the included patients, there were 26 males and 37 females. The primary tumor locations included the lower extremities in 36 cases, upper extremities in 8 cases, and the trunk in 19 cases (Fig. 1). The demographic of patients showed a higher distribution in females (male-to-female ratio of 1:1.42) and a wide age range (5–68 years), but mainly concentrated in young people, with an average age of 25.9 ± 12.0-year-old (Table 1).

**Table 1:** Descriptive statistics and distribution of clinical features according to tumor histological grade

Characteristics	ASPS	Low-grade group, (*n* = 29)	High-grade group, (*n* = 34)	*p* value
Gender				0.120
Male	26/63 (41.3%)	15/29 (51.7%)	11/34 (32.4%)	
Female	37/63 (58.7%)	14/29 (42.3%)	23/34 (67.6%)	
Age				0.572
< 30-year-old	35/63 (55.6%)	15/29 (51.7%)	20/34 (58.8%)	
≥ 30-year-old	28/63 (44.4%)	14/29 (42.3%)	14/34 (41.2%)	
Location				0.951
Upper limb	8/63 (12.7%)	4/29 (13.8%)	4/34 (11.8%)	
Lower limb	36/63 (57.1%)	16/29 (55.2%)	20/34 (58.8%)	
Trunk	19/63 (30.2%)	9/29 (31.0%)	10/34 (29.4%)	
Depth				0.115
Deep	39/63 (61.9%)	18/29 (62.1%)	21/34 (61.8%)	
Superficial	18/63 (28.6%)	13/29 (44.8%)	5/34 (14.7%)	
Deep and superficial	6/63 (9.5%)	2/29 (6.9%)	4/34 (11.8%)	
Size (cm)				0.094
< 5	18/63 (31.1%)	12/29 (42.9%)	6/34 (20.9%)	
5–10	21/63 (35.6%)	9/29 (38.1%)	12/34 (33.3%)	
≥ 10	24/63 (33.3%)	8/29 (19.0%)	16/34 (45.8%)	
Distant metastasis				0.031^a^
No	32/63 (44.4%)	19/29 (64.7%)	13/34 (26.3%)	
Yes	31/63 (55.6%)	10/29 (35.3%)	21/34 (73.7%)	
CD34 expression				0.712
Negative	16/63 (25.4%)	8/29 (27.6%)	8/34 (23.5%)	
Positive	47/63 (74.6%)	21/29 (72.4%)	26/34 (76.4%)	
TFE3 expression				0.858
Weakly positive	21/63 (33.3%)	10/29 (34.5%)	11/34 (32.4%)	
Strongly positive	42/63 (66.7%)	19/29 (65.5%)	23/34 (67.6%)	
Ki-67 expression (%)				0.004^a^
< 30	31/63 (49.2%)	20/29 (69.0%)	11/34 (32.4%)	
≥ 30	32/63 (50.8%)	9/29 (31.0%)	23/34 (67.6%)	

^a^ *p* indicates that the difference is statistically significant

#### Metastatic patterns

The metastatic spread of ASPS was observed in a considerable portion of patients. Lung metastasis was the most common (27 cases), followed by bone, lymph node, and brain metastases. Additionally, a subset of patients presented with multiple systemic metastases (16 cases).

#### Tumor size and shape

The tumors in this cohort exhibited a wide range of sizes, the maximum tumor diameter ranged from 23 mm to 151 mm, with an average of (82.53 ± 34.13) mm (Table 2).

**Table 2:** Descriptive statistics and distribution of MRI features according to tumor histological grade

Characteristics	Low-grade group, (*n* = 29)	High-grade group, (*n* = 34)	*p* value
Shape			0.549
Round	11/29 (37.9%)	15/34 (44.1%)	
Irregular	18/29 (62.1%)	19/34 (55.9%)	
Tumor boundary			0.179
Clear	16/29 (55.2%)	13/34 (26.3%)	
Unclear	13/29 (44.8%)	21/34 (73.7%)	
Peritumoral edema			0.923
No	15/29 (51.7%)	18/34 (52.9%)	
Yes	14/29 (42.3%)	16/34 (47.1%)	
Intratumoral necrosis			0.037^a^
No	17/29 (58.6%)	11/34 (32.4%)	
Yes	12/29 (41.4%)	23/34 (67.6%)	
T1WI signal			0.360
Equal/low	12/29 (42.9%)	18/34 (52.9%)	
Slightly high	17/29 (57.1%)	16/34 (47.1%)	
T2WI signal			0.120
Iso/low	15/29 (51.7%)	11/34 (32.4%)	
Mixed high	14/29 (42.3%)	23/34 (67.6%)	
DWI signal			0.297
Equal/slightly high	14/29 (42.3%)	12/34 (35.3%)	
High	15/29 (51.7%)	22/34 (64.7%)	
Enhancement characteristics			0.034^a^
Uniform enhancement	18/29 (62.1%)	12/34 (35.3%)	
Uneven enhancement	11/29 (37.9%)	22/34 (64.7%)	
TIC type			0.020^a^
Type I	4/29 (13.8%)	0/34 (0%)	
Type II	12/29 (41.4%)	9/34 (26.5%)	
Type III	13/29 (44.8%)	25/34 (73.5%)	
ADC mean ( × 10^−3^ mm^2^/s)	1093.7 (986.4, 1370.8)	964.5 (800.0, 1049.7)	0.002^a^

*DWI* diffusion-weighted imaging, *ADC* apparent diffusion coefficient

^a^ *p* indicates that the difference is statistically significant

#### MRI features

**Fig. 2:**
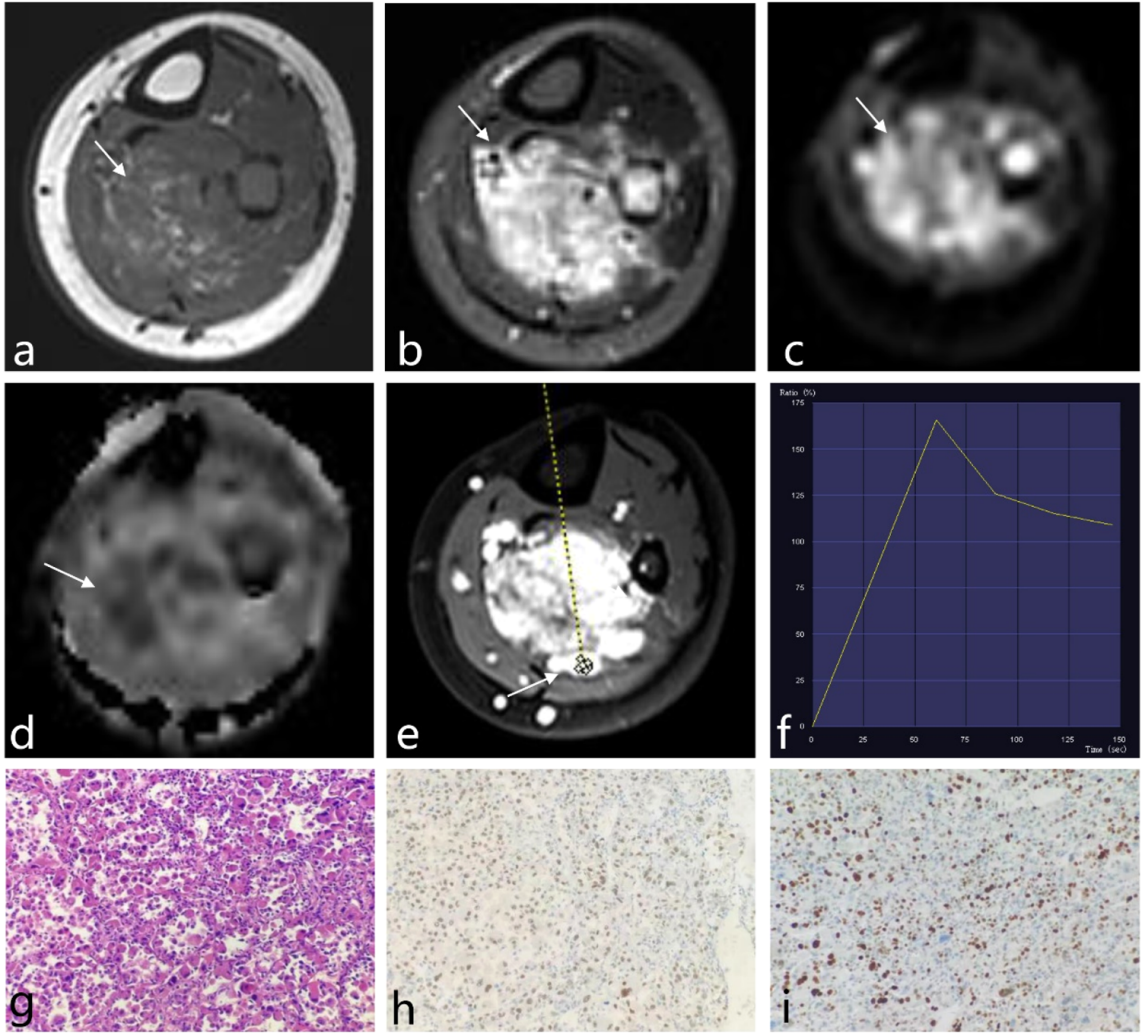
**a** Five-year-old female with an irregular mass in the left posterior compartment of the lower leg. Postoperative pathology revealed high-grade ASPS. **a**–**e** Depict transverse axis MRI images of the mass: (**a**) T1WI showed a heterogeneous high signal, (**b**) fat-suppressed T2WI displayed a markedly high signal, (**c**) DWI exhibited a high signal, (**d**) ADC mapping showed a mixed high and low signal, and (**e**) post-contrast T1WI revealed significant heterogeneous enhancement. The TIC in **f** demonstrated a rapid rise followed by a rapid decline (Type III curve). In the histopathological examination (**g**), cells were organized in an organoid or adenoid cystic pattern, with multiple fibrous septa visible between cells (Hematoxylin and Eosin stain, × 200). Immunohistochemistry in **h** demonstrated diffuse nuclear positivity for TFE3. Ki-67 expression in **i** was approximately 40%

**Fig. 3:**
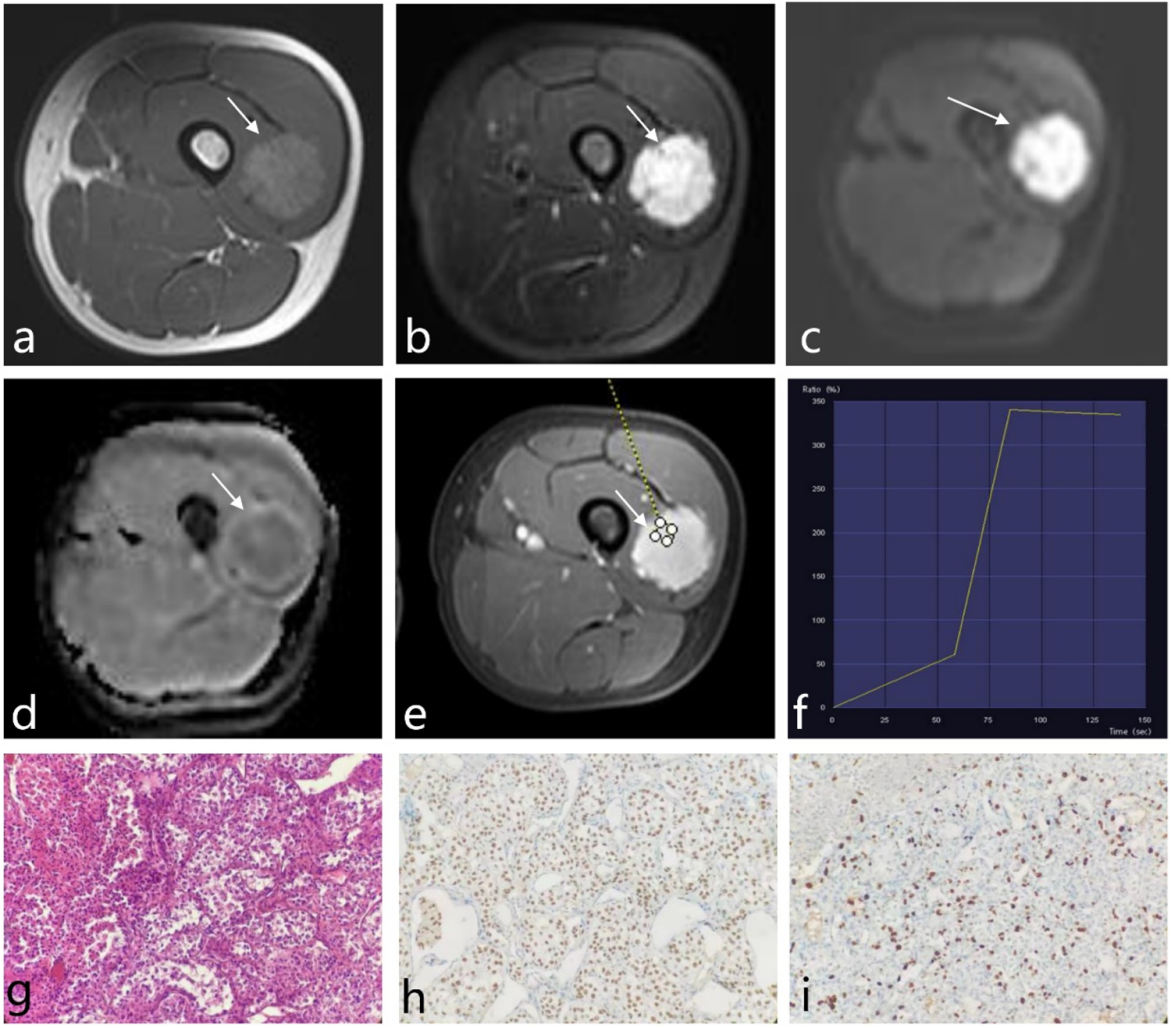
**a** Thirty-year-old female with a circular mass located in the inner aspect of the left thigh’s lateral muscle group. Postoperative pathology revealed low-grade ASPS. **a**–**e** Show transverse axis MRI images of the mass: (**a**) T1WI displayed uniformly slightly high signal, (**b**) fat-suppressed T2WI exhibited markedly high signal, (**c**) DWI showed high signal, (**d**) ADC mapping demonstrated central low signal with slightly high signal at the periphery, and (**e**) post-contrast imaging revealed significant and uniform enhancement. The TIC in **f** demonstrated a rapid rise followed by a plateau (Type II curve). In the histopathological examination (**g**), cells were organized in an organoid or adenoid cystic pattern, with mostly polygonal epithelioid cells and multiple fibrous septa visible between cells (Hematoxylin and eosin stain, × 200). Immunohistochemistry in **h** showed diffuse nuclear positivity for TFE3. Ki-67 expression in **i** was approximately 15%

High-grade ASPS tumors typically displayed several distinct radiological characteristics, such as larger size, unclear margins, Type III washout on TIC analysis, and mixed low signal on ADC maps (Table 2). Figures 2a–f and 3a–f showed typical MRI features in patients with high-grade and low-grade ASPS, respectively.

#### Histological characteristics

Histologically, ASPS tumors displayed characteristic features, including large polygonal cells with prominent nuclei and abundant cytoplasm. These cells formed distinctive alveolar or nest-like structures that were densely packed and often surrounded by delicate, thin-walled sinusoidal-like blood vessels (Figs. 2g and 3g).

#### Molecular markers

Immunohistochemical analysis of TFE3 gene expression revealed a wide range of staining intensities. Weakly positive staining was observed in 21 cases, while 42 cases showed strong positivity (Figs. 2h and 3h). Ki-67, a marker of cellular proliferation, exhibited a median expression of 37.5 (21.25, 53.75) percent in the high-expression group (Fig. 2i) and 20.0 (15, 30) percent in the low-expression group (Fig. 3i).

#### Consistency of imaging findings

Based on the results of two observers employing a double-blind method to analyze conventional MRI features (location, morphology, size, margins, peritumoral edema, intratumoral necrosis, T1WI signal, T2WI signal, DWI signal, and tumor enhancement degree) and DWI parameters (ADC values), the ICC values all exceeded 0.74, showing excellent consistency.

### Comparative analysis of clinical and pathological data of ASPS patients with different histological grades

Analysis of all clinical and pathological information revealed statistically significant differences between the high-grade and the low-grade groups with respect to distant metastasis and Ki-67 expression (*p* values were 0.031 and 0.004, respectively) (Table 1).

### Comparative analysis of MRI features of ASPS patients with different histological grades

As shown in Table 2, among all MRI features, differences in intratumoral necrosis, tumor enhancement characteristics, TIC type, and ADCmean were statistically significant between the high-grade and the low-grade groups (*p* < 0.05). The ADC values in the high-grade group were significantly lower than those in the low-grade group (*p* = 0.002).

Binary logistic regression analysis was performed for distant metastasis, intratumoral necrosis, enhancement characteristics, TIC type, and ADC mean. The results indicated that distant metastasis (*p* = 0.016, OR = 0.176, 95%CI 0.043–0.721), enhancement characteristics (*p* = 0.028, OR = 0.221, 95% CI 0.057–0.852), and ADC values (*p* = 0.007, OR = 0.995, 95% CI 0.992–0.999) were independent predictors of high-grade tumors.

### Correlation analysis of ADC values with histological grade and Ki-67 expression

**Fig. 4:**
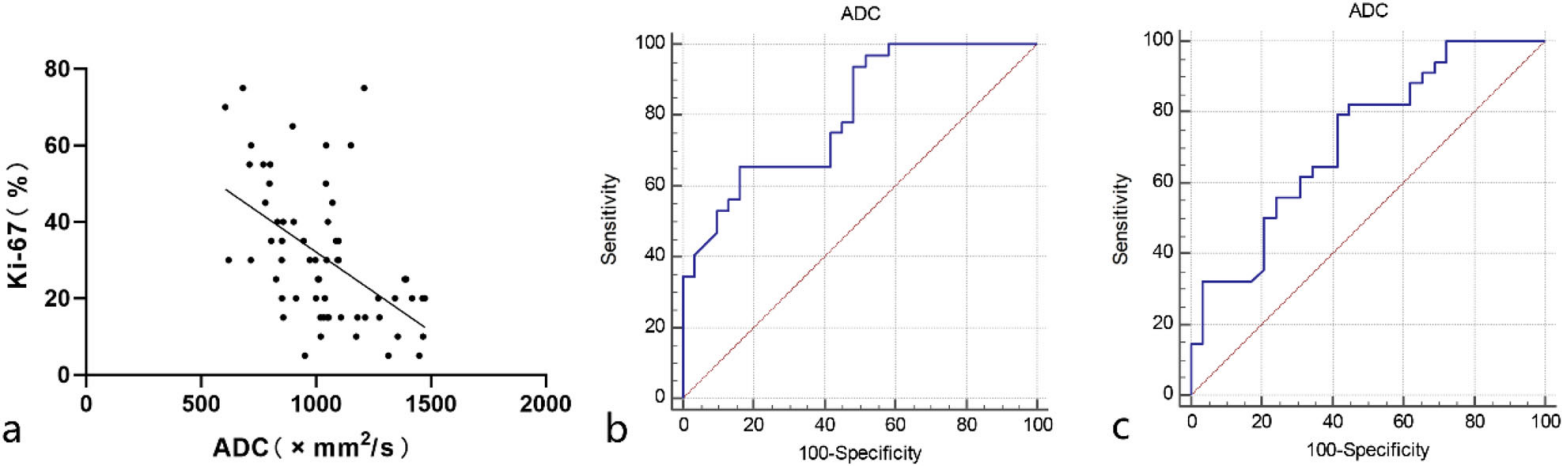
Scatterplot and ROC analysis of ADC values with Ki-67 expression. There was a moderate negative correlation between ADC values and Ki-67 expression (**a**). The AUC for discriminating between high and low Ki-67 expression based on ADC values was 0.805 (**b**), and for distinguishing between high-grade and low-grade ASPS groups was 0.723 (**c**)

Spearman correlation analysis revealed a negative correlation between ADC values and Ki-67 expression (*r* = −0.526; *p* < 0.001) (Fig. 4a). ROC analysis showed that when the ADC threshold value was 0.997 × 10^−3^ mm^2^/s, the AUC, sensitivity, and specificity for distinguishing between high and low Ki-67 expression were 0.805, 65.6%, and 83.9%, respectively (Fig. 4b). When the ADC threshold value was 1.052 × 10^−3^ mm^2^/s, the AUC, sensitivity, and specificity for distinguishing between high-grade and low-grade ASPS were 0.723, 79.4%, and 58.6%, respectively (Fig. 4c).

## Discussion

Our study highlights the clinical, MRI, and pathological manifestations of ASPS and the important role of MRI features in distinguishing between high- and low-grade ASPS. The differentiation is critical, given the insidious nature of ASPS, which often presents as asymptomatic, with early metastasis and resistance to conventional chemotherapy. The correlation we observed between qualitative MRI features (distant metastasis, intratumoral necrosis, and tumor enhancement), semi-quantitative features (TIC type), and quantitative features (ADC values) with histological grades and Ki-67 expression levels offers a new avenue for non-invasive preoperative assessment. This is particularly important in the context of ASPS, where conventional biopsy techniques present risks and limitations due to tumor heterogeneity and potential for cell dissemination.

ASPS is a rare, malignant, highly vascularized tumor with distinctive morphological features that differ from other STSs, it exhibits some typical clinical, pathological, and MRI characteristics. It predominantly affects young to middle-aged females, characterized by characteristic nest-like structures separated by fibrous connective tissue rich in blood sinuses, and cells arranged in a “glandular” or “organ-like” pattern [3]. In our study, the nest-like or glandular structures and sinus-like blood vessels within tumors were observed. Due to its rich vascularity and slow blood flow, these tumors often display multiple tortuous blood vessels within and around the mass, resulting in slightly increased signal intensity on T1WI and high signal intensity on DWI, which is in line with our study results [12].

ASPS typically shows moderate or marked enhancement on contrast-enhanced scans due to its rich vascularity. Qiao et al [13] analyzed 14 cases of ASPS and found that they all exhibited significant enhancement on contrast-enhanced scans. In our study, we found that 19 patients showed marked enhancement compared to the tumor T1WI signal, especially in high-grade ASPS patients, which is consistent with literature reports. The TIC as a semi-quantitative parameter of DCE-MRI can comprehensively reflect vascular permeability, perfusion, and microcirculation status. Malignant lesions typically exhibit abundant blood flow perfusion, appearing as rapid peak enhancement on the TIC curve, whereas benign lesions often display flat TIC curves [12, 14, 15]. Several studies on musculoskeletal tumors have shown that contrast-enhanced scans are helpful in differentiating between benign and malignant lesions, with Type III TIC curves often indicating malignant tumors, but both benign and malignant lesions can present as Type I or Type II curves [14–16]. In our study, we found that TIC types in ASPS helped distinguish between high and low grades, with Type III curves being predominant, reflecting that higher-grade tumors have a richer capillary network, consistent with the feature of ASPS being highly vascularized. Our findings regarding the relationship between MRI characteristics and tumor grade further underscore the potential of MRI in providing insights beyond basic diagnostic imaging. For instance, the significant enhancement patterns observed in contrast-enhanced scans reflect ASPS’s rich vascularity, aligning with previous studies and suggesting that MRI can capture the unique vascular profile of these tumors.

Moreover, the ability of MRI to identify distant metastases, a predictor of high-grade ASPS, underscores its potential in early detection and staging, which is crucial for effective treatment planning. ASPS patients are often asymptomatic, and distant metastasis may have already occurred at the time of diagnosis [1–3]. In our study, 31 cases had distant metastases at the initial diagnosis, involving the lungs, brain, bones, and lymph nodes. We found that the high-grade group had a significantly higher metastasis rate than the low-grade group, and distant metastasis was an independent predictor of high-grade ASPS. The rich blood supply in ASPS, along with the presence of large, tortuous blood vessels within and around the tumor, may be important factors contributing to early distant metastasis [13–16]. In previous studies, tumor necrosis was considered a reliable predictor of high-grade STS, and for STS patients with necrosis volume less than 50%, necrosis volume exceeding 50% indicated a worse prognosis [17, 18]. In our study, we found that high-grade ASPS exhibited more necrosis, consistent with literature reports.

DWI reflects the rate of diffusion of water molecules, and the typical feature of malignant tumors is high cell density and reduced extracellular space, resulting in restricted water molecule diffusion and consequently lower ADC values. Chhabra et al [19] found that tumor size, central enhancement, and ADC values could be used to differentiate the histological grade of musculoskeletal soft tissue malignant tumors. In our study, we found that the ADC values in high-grade ASPS tumors were lower than those in low-grade tumors. The positive correlation between cell density and tumor grade can explain the ability of DWI to assess ASPS histological grade. In breast cancer, hepatocellular carcinoma, cervical cancer, and other solid tumors, ADC values have been reported to be negatively correlated with Ki-67 expression [20–22]. Lee et al [23] first found a weak negative correlation between ADC values and Ki-67 expression in STS, and in subsequent reports, Yuan et al [24] found a moderate negative correlation between ADC and D values and Ki-67 expression in a rhabdomyosarcoma mouse model. Fang et al [25] found that histogram features such as ADCmean and ADCmin were negatively correlated with Ki-67 expression in fibrosarcoma. In our study of ASPS, the observation of a negative correlation between ADC values and Ki-67 expression levels further supports the use of MRI in the non-invasive assessment of tumor aggressiveness and proliferation activity. This correlation not only validates the utility of MRI in differentiating tumor grades but also opens avenues for its use in monitoring treatment response and potentially guiding therapy adjustments.

While our study establishes a strong correlation between MRI characteristics and ASPS histological grades, it also raises important considerations regarding the specificity and stability of qualitative diagnosis in different STS subtypes. The varying manifestations of conventional MRI features like peritumoral enhancement and edema, which did not predict high-grade ASPS in our study, suggest the need for a more nuanced understanding of MRI readings in different soft tissue sarcoma contexts. Furthermore, our findings on the molecular characteristics of ASPS, particularly the expression of TFE3, add a layer of complexity to the disease’s diagnostic landscape. Although our study showed universal positive expression of TFE3 across all samples, the lack of significant statistical difference in histological grade raises questions about the marker’s predictive power, possibly limited by our study’s small sample size.

Histopathological confirmation provides precise evidence of the disease and detailed biological insights, while MRI offers crucial visual information regarding tumor anatomical location, size, relationship with surrounding tissues, and treatment response. Given that tumor heterogeneity can impact histological diagnosis from percutaneous biopsies, MRI serves as an additional non-invasive diagnostic tool, thereby complementing the limitations of biopsies. Our study indicates that MRI features such as distant metastases, heterogeneous tumor enhancement, and low ADC values (< 1.052 × 10^−3^ mm^2^/s) contribute to the diagnosis of high-grade ASPS. Additionally, when observing an ADC less than 0.997 × 10^−3^ mm^2^/s, high expression of Ki-67 suggests a poorer prognosis for patients. These insights aid physicians in determining the nature of the tumor, and evaluating tumor enhancement characteristics, growth rate, and prognosis, thus providing crucial evidence for the selection of treatment modalities and prognosis assessment for ASPS patients [23, 26, 27]. Our study indicated that high-grade ASPS typically exhibits lower ADC values, and ADC values are inversely correlated with Ki-67 expression levels. Therefore, ADC values can serve as an important indicator for predicting the malignancy and prognosis of ASPS. Based on ADC values, physicians can more accurately assess the severity of ASPS and devise personalized treatment plans. In summary, MRI examination results have a significant impact on the clinical decisions and management strategies for ASPS patients.

Several limitations should be considered. Firstly, this study involved a relatively small number of ASPS patients, which may impact the generalizability and reliability of the results. Expanding the sample size could contribute to a more comprehensive understanding of ASPS characteristics. Secondly, due to the heterogeneity of tumors, measuring ADC only in the largest lesion slice may lead to misdiagnosis. Thirdly, the results of this study were applicable only to cases with known histological diagnoses, lacking applicability. For situations where histological diagnosis has not been conducted or diagnostic uncertainty exists, these results may not be effectively applicable. Lastly, this study primarily focused on initial diagnosis-related features and predictive factors, but lacked long-term follow-up data for patients, making it impossible to assess treatment outcomes and survival rates.

In conclusion, our study underscores the MRI manifestations of ASPS and the vital role of MRI in the preoperative assessment of ASPS, providing clinicians with valuable information on tumor diagnosis, grading, and proliferation activity. By leveraging both conventional MRI and DWI, we can enhance our understanding of this rare tumor’s characteristics, aiding in the development of tailored treatment strategies.

## Data Availability

Data available on request: the data underlying this article will be shared on reasonable request to the corresponding author.

## Abbreviations

ADC: Apparent diffusion coefficient
ASPS: Alveolar soft part sarcoma
AUC: Area under the curve
DCE: Dynamic contrast-enhanced
DWI: Diffusion-weighted imaging
ICC: Intraclass correlation coefficient
MRI: Magnetic resonance imaging
ROC: Receiver operating characteristic
TIC: Time-intensity curve
